# Activation of cancer immunotherapy by nanomedicine

**DOI:** 10.3389/fphar.2022.1041073

**Published:** 2022-12-22

**Authors:** Lijuan Wang, Henan Xu, Lili Weng, Jin Sun, Ye Jin, Chunping Xiao

**Affiliations:** ^1^ College of Pharmacy, Changchun University of Chinese Medicine, Changchun, China; ^2^ The First Hospital of Jilin University, Changchun, China

**Keywords:** immunotherapy, the CAR-T therapy, nanoparticles, drug delivery, nanoparticles combined with CAR-T therapy

## Abstract

Cancer is one of the most difficult diseases to be treated in the world. Immunotherapy has made great strides in cancer treatment in recent years, and several tumor immunotherapy drugs have been approved by the U.S. Food and Drug Administration. Currently, immunotherapy faces many challenges, such as lacking specificity, cytotoxicity, drug resistance, etc. Nanoparticles have the characteristics of small particle size and stable surface function, playing a miraculous effect in anti-tumor treatment. Nanocarriers such as polymeric micelles, liposomes, nanoemulsions, dendrimers, and inorganic nanoparticles have been widely used to overcome deficits in cancer treatments including toxicity, insufficient specificity, and low bioavailability. Although nanomedicine research is extensive, only a few nanomedicines are approved to be used. Either Bottlenecks or solutions of nanomedicine in immunotherapy need to be further explored to cope with challenges. In this review, a brief overview of several types of cancer immunotherapy approaches and their advantages and disadvantages will be provided. Then, the types of nanomedicines, drug delivery strategies, and the progress of applications are introduced. Finally, the application and prospect of nanomedicines in immunotherapy and Chimeric antigen receptor T-cell therapy (CAR-T) are highlighted and summarized to address the problems of immunotherapy the overall goal of this article is to provide insights into the potential use of nanomedicines and to improve the efficacy and safety of immunotherapy.

## 1 Introduction

Cancer has become the second fatal disease in the world after cardiovascular diseases, and the number of patients is increasing year by year (Mullard 2020). Current tumor treatments methods mainly include surgery, radiotherapy, chemotherapy, and targeted drug therapy. Conventional surgical resection is difficult to remove the tumor cells completely, so it is easily leading to a postoperative recurrence. Radiotherapy and chemotherapy can kill tumor cells as well as normal cells, causing more harm to patients. Although targeted drug therapy can reduce the side effects of drugs, it is prone to cause tumor resistance and tumor recurrence. With the development of tumor biology, immunotherapy has emerged as a powerful clinical strategy for cancer. Different from conventional treatments, tumor immunotherapy targets mainly immune cells by activating the body’s immune system, achieve the elimination of tumor cells. It has the advantages of good effects, low adverse, and prevention of recurrence (Tan et al., 2020; Zhang and Zhang 2020). Over the past decade, there have been significant developments in the treatment of cancer. The introduction of immunotherapies such as immune checkpoint inhibitors, cellular immunotherapy, antibody therapy, and cytokine therapy help, patients tackle malignancies by boosting immune systems. Unfortunately, as immunotherapy continues to evolve, its side effects have emerged, including off-target effects, whose reason can be the complexity of treatment process, and the viability loss of T cells (Singh and McGuirk 2020; Sterner and Sterner 2021). Although some can be reversed by the use of immunomodulators (such as corticosteroids), high morbidity and mortality still occurs due to other reasons (Kiesgen et al., 2021). Many patients present other symptoms, such as pseudoprogression, hyperprogression, isolated responses, and drug resistance. Despite the rate of pseudoprogression and hyperprogression are not high, related reports are increasing, while immunotherapy is widely used.

Nano drug is a new way cancer immunotherapy with a safer and more controlled manner (Martin et al., 2020), allowing the expansion of therapeutic agents to more patients and reducing toxicity. In particular, nanotechnology can accumulate the efforts of immunotherapy in diseased tissues, desirable tumor and/or immune cells effectively and reduce the negative effects of off-targeting (Qiu et al., 2017). Moreover, the feature of nanomaterials such as temperature-sensitive properties and pH sensitivity can be used to improve therapeutic tolerance while enhancing targeting (Li et al., 2011; Cifuentes-Rius et al., 2021). By optimizing the morphological characteristics and surface properties (Xu et al., 2012), nano drug can have a prolonged half-life in the reticuloendothelial system circulation (Li et al., 2012). Therefore, nanomedicines in immunotherapy are considered as promising strategies for treating and even curing certain types of cancer. Researchers are currently investigate the development of novel nanomedicines for immunotherapy, including nanoparticles, nanocouples, and biomaterials (Shields et al., 2020; Brouillard et al., 2021).

Among cellular immunotherapies chimeric antigen receptor T-cell therapy (CAR-T) has become the focus of clinical immuno-oncology because of its MHC-independence, broad-spectrum replicability and immune memory function (Kiesgen et al., 2021). This therapy targets refractory or relapses B-cell precursor acute lymphoblastic leukemia in a target group of patients mainly under 25 years (Dai et al., 2020; Lu et al., 2020). There are various treatments for B-cell lymphoma, including chemotherapy, small molecule inhibitors of the B-cell receptor pathway, antibody-drug couples, immune checkpoint inhibitors, and RNA interference. Different from these approaches, CAR-T therapies isolate T-cells from a patient’s blood with genetical engineering techniques to express a CAR bindings to B-cell CD19. After *in vitro* amplification, cells are injected back into the patient, and then continue to proliferate and find target cells *in vivo* (Wei et al., 2020). To simplify the CAR-T treatment approach, Stephan’s group has developed a low-cost technology based on gene delivery. Polymeric nanoparticles targeting T-cells were prepared by combining leukemia-specific 194-1BBz CAR plasmid DNA with poly-β amino esters. Nanoparticles were covered with anti-CD3e f (ab’) two fragment modified polyglutamic acid by electrostatic interactions. To achieve a rapid nuclear localization of gene drugs (Manchanda et al., 2021), the nanoparticles were further modified with peptides containing nuclear localization signals. *In vitro*, it was observed that the nanoparticles were rapidly taken up by T-cells within 2 h. After 30 h of incubation, about 4% of T-cells were detected as CAR+, which demonstrates a significant effect (Kapadia et al., 2015). In addition to the complexity of the therapeutic process, CAR-T therapies are also prone to loss of viability of their effector T cells rapidly due to the host’s immunosuppressive microenvironment. Certain cytokines can enhance the viability and function of effector T-cells, however, they can also cause serious side effects. Bai’s group (Bai et al., 2020) found that self-assembled multivalent nucleic acid aptamer nanoparticles with CAR-like properties could increase T-cell proliferation and reverse the inhibitory effect of exogenous B7.1 molecules on Interleukin-2 (IL-2) secretion *in vitro*. The restriction of melanoma B16 cells growth in mice could be achieved both *in vitro* and *in vivo*, demonstrating that T-cells activated by multivalent aptamer nanoparticles could achieve the same function of CAR-T, enhancing the safety of CAR-T cell immunotherapy. Therefore, some shortcomings of pure CAR-T therapy can be overcome with the usage of nanomedicines.

## 2 Immunotherapy

Tumor immunotherapy is a sort of cancer treatments by activating the immune system (Dai et al., 2020), which has become a new tool for tumor treatment. Compared with conventional treatments, tumor immunotherapy mainly targets immune cells and activate body’s immune system by inhibiting immune negative regulatory factors and enhancing the recognition ability of immune cells to the surface antigens of tumor cells, and achieve the elimination of tumor cells. Up till now, tumor immunotherapy has evolved into various forms, including immune checkpoint inhibitors, antibody therapy, cellular immunotherapy, cytokine therapy, and immune combination therapy (Kumar et al., 2021). Therefore, there are many marketed drugs of immunotherapy (Table 1).

**Table 1:** Summary of marketed drugs of immunotherapy.

Pharmaceuticals	Characteristics	TTM
Amphotericin B	Polyene antifungal antibiotics inhibit fungal growth by affecting cell membrane permeability	1958
Vinorelbine tartrate micelles for injection	By inhibiting the polymerization of kinetochore tubulin, it stops cell division at metaphase of mitosis, a cell cycle specific drug	1989
Leuprorelin	Gonadotrophins, villous solids, commonly used in endometriosis	1994
Octreotide	A synthetic octapeptide circular compound that has a similar effect to that of native endogenous somatostatin but is stronger and more persistent, with a half-life 30 times longer than that of native somatostatin	2003
Gefitinib	A selective epidermal growth factor receptor (EGFR) tyrosine kinase inhibitor that inhibits tumor cell growth and increases apoptosis in tumor cell-derived lines. It can improve the antitumor activity of chemotherapy, radiotherapy and hormone therapy, and is a small molecule targeted drug for systemic targeted therapy of tumors	2003
Bevacizumab	A recombinant humanized immunoglobulin G1 (IgG1) monoclonal antibody can bind VEGF-A to inhibit its binding to VEGF receptor-2 (VEGFR-2), and then inhibit the biological effects of VEGF, including affecting vascular permeability, proliferation and endothelial cell migration and survival, so as to inhibit tumor angiogenesis, growth and metastasis	2004
Erlotinib	A highly specific and reversible inhibitor of epidermal growth factor receptor tyrosine kinase, primarily used in the second - and third-line treatment of non-small cell lung cancer after chemotherapy failure	2005
Crizotinib	The efficacy of ALK and ROS1 targeted agents against both mutations was significantly better than that of conventional chemotherapy, and the safety and tolerability were higher	2011
Lcotinib	A highly potent and specific epidermal growth factor receptor tyrosine kinase inhibitor (EGFR-TKI). In the screening of 85 kinases, icotinib could selectively inhibit EGFR and its 3 mutants, but had no significant inhibitory effect on the remaining 81 kinases	2011
CimaVax	The vaccine, made up of two proteins, epidermal growth factor and P64K, is injected into the body to stimulate the immune system of patients with NSCLC to produce corresponding antibodies. The antibody removes epidermal growth factor from the patient’s body, depriving lung cancer cells of nutrients to grow and eventually go into apoptosis	2011
Ipilimumab (Yervoy)	A monoclonal antibody that effectively blocks a molecule called cytotoxic T-cell antigen-4. Ctla-4 affects the body’s immune system, impairing its ability to kill cancer cells	2011
Afatinib	It is a potent and irreversible dual inhibitor of epidermal growth factor receptor (EGFR) and human epidermal growth factor receptor 2 (HER2) tyrosine kinase	2013
Ramucirumab	Angiogenesis inhibition, vascular endothelial growth factor-2 (VEGFR-2) is the specific target of ramirumab, its 50% maximum inhibitory concentration is 0.8–1.0 nM	2014
Ceritinib	Tyrosine kinase inhibitor and cytochrome P4503A inhibitor and cytochrome P4502C9 inhibitor	2014
Nivolumab	A monoclonal antibody against PD1 used as an adjuvant therapy in patients with melanoma with lymph node metastases	2014
Pembrolizumab	A PD-(L)1 tumor immunotherapy, by targeting the blockade of the PD-1/PD-L1 pathway, utilizes the body’s own immune system to fight tumors, and has the potential to treat many types of tumors	2014
Leuprolide acetate microspheres	It has a certain inhibitory effect on the pituitary gonad, and has obvious therapeutic effect on prostate cancer, endometriosis, uterine fibroids and other related sex hormones, related diseases or tumors	2015
Osimertinib	A small molecule targeted antitumor agent for the treatment of non-small cell lung cancer, is a third representative dermal growth factor receptor inhibitor	2015
Neci tumumab	A recombinant human monoclonal antibody to lgG1 that binds to the human epidermal growth factor receptor (EGFR), thereby blocking the binding of EGFR to its ligand	2015
Alectinib	Tyrosine kinase inhibitors of ALK and RET, the principle of action is to inhibit the phosphorylation of ALK and the activation of signaling proteins, and can continuously inhibit the proliferation of cancer cells and the mutation of ALK, and eventually slow down the progression of cancer	2015
Atezolizumab	A monoclonal antibody that targets the PD-L1 protein. Attegizumab binds to PD-L1 expressed on tumor cells and tumor infiltrating immune cells, blocking its interaction with PD-1 and B7.1 receptors. By inhibiting PD-L1, it can activate T-cells to destroy tumor cells	2016
Avelumab	A humanized monoclonal antibody that acts as a programmed cell death ligand (PD-L1) blockade agent that binds to PD-L1 on tumor cells and blocks its interaction with T-cells and antigen presenting cell PD-1, thereby relieving PD-1/PD-L1-mediated immune suppression and promoting T-cells to attack tumor cells	2017
Durvalumab	A human immunoglobulin G1κ(IGG1κ) monoclonal antibody that blocks the interaction of programmed cell death ligand 1(PD-L1) with PD-1(CD279)	2017
Brigatinib	A unique structural feature is an neglected phosphine oxide, a novel hydrogen bond acceptor that drives efficacy and selectivity in addition to good ADME performance	2017
Dabrafenib	A BRAF protein kinase retarder for internal administration	2017
Vemurafenib	A low molecular weight oral inhibitor of BRAF serine-threonine kinase mutants, including BRAF V600E. Vemurafenib also inhibited other kinases at similar concentrations *in vitro*, such as CRAF, ARAF, wild-type BRAF, SRMS, ACK1, MAP4K5, and FGR.	2017
Trametinib	Mitogen-activated protein kinase (MAPK) pathway can transmit signals from active extracellular receptors to intracellular receptors	2017
Larotrectinib	Highly specific oral TRK inhibitors that target specific genetic mutations rather than specific cancer types	2018
Dacomitinib	The efficacy was very similar for the two major EGFR mutation types: exon 19 deletion and exon 21 mutation	2018
Lorlatinib	Tyrosine kinase inhibitors (TKI) are the third generation of small molecule targeted drugs targeting ALK mutations, which can selectively bind the products of ALK and ROS1 fusion driver gene variants, block the activation and transmission of downstream signaling pathways, and thus inhibit the growth and proliferation of cancer cells with these mutations, and play an anti-cancer role	2018
Toripalimab	It is indicated for the treatment of unresectable or metastatic melanoma that has failed previous systemic therapy	2018
Sintilimab	Programmed Cell Death 1 (PD-1) is a recombinant human immunoglobulin G (IgG4) type monoclonal antibody Programmed Cell Death 1 (PD-1). By binding PD-1 and blocking the binding of PD-1 to PD-L1 and PD-L2, the immunosuppressive effect is lifted and the function of T-cells is activated. Enhance the immune surveillance and killing ability of T-cells to tumor, and generate tumor immune response	2018
Cemiplimab	By specifically binding to PD-1, LIBTAYO can “break” the inhibition of T cells by cancer cells, and T-cell receptor signals can be reactivated to restore anti-tumor activity, so as to enhance the killing ability of human immune system to cancer cells and achieve anti-cancer effect	2018
Pembrolizumab	Binding of PD-1 ligands (PD-L1 and PD-L2) to PD-1 receptors on T-cells inhibits T-cell proliferation and cytokine production. Pd-1 ligand is up-regulated in some tumor cells, which can inhibit the monitoring of tumor by active immune T-cells through this signaling pathway	2018
Camrelizumab	A humanized anti-PD-1 monoclonal antibody can bind to the human PD-1 receptor and block the PD-1/PD-L1 pathway to restore the body’s anti-tumor immunity, thus forming the basis of cancer immunotherapy	2019
Tislelizumab	An investigational human monoclonal antibody that belongs to a class of tumor immune drugs known as immune checkpoint inhibitors	2019
Entrectinib	Oral tyrosine kinase inhibitors with central nervous system activity primarily target tumors with NTRK1/2 2/3,ROS1, or ALK gene fusion mutations	2019
Selpercatinib	Inhibition of native RET signaling and the expected mechanisms of acquired resistance in patients with tumors carrying abnormal RET kinases	2020
Capmatinib	A kinase inhibitor that targets MET, including mutated variants produced by exon 14 hopping. The jump in MET exon 14 results in the loss of a protein regulatory structure that reduces its negative regulation and thus increases downstream MET signaling	2020
Furmonertinib	An Epidermal Growth factor receptor Tyrosine kinase inhibitor (EGFR-TKI)	2021
amivantamab-vmjw Rybrevant	A bisecific antibody to whole human EGFR-mesenchymal transforming factor (MET) with immune cell-directed activity targeting tumors carrying activated and resistant EGFR and MET mutations and expansions	2021
Sotorasib	One of the first small molecule inhibitors to successfully target KRAS and enter human clinical development, targeting KRAS protein carrying the G12C mutation. Sotorasib specifically and irreversibly inhibits the pro-proliferative activity of G12C mutant KRAS protein by locking it into a non-activated GDP-binding state	2021
Pralsetinib	Kinase inhibitors of wild-type RET and oncogenic RET fusion (CCDC6-RET) and mutation (RET V804L, RET V804M, and RET M918T)	2021
Savolitinib	It can selectively inhibit the phosphorylation of MET kinase and significantly inhibit the proliferation of tumor cells with MET exon 14 hopping	2021
Tepotinib	A kinase inhibitor targeting MET, including the METex14 hopping mutation. Tepotinib, administered once daily, inhibits MET phosphorylation and subsequent downstream signaling pathways to inhibit tumor growth, anchor-independent growth, and MeT-dependent tumor cell migration	2021
Polymer micelles of paclitaxel for injection	Through passive targeting, the drug is introduced into the tumor microenvironment with vascular disorder. In this way, the drug is more likely to stay in the tumor tissue, resulting in high concentration in the tumor tissue and low concentration in the normal tissue. While improving the efficacy, the incidence of toxic and side effects is further reduced	2021
Penpulimab	A novel PD-1 monoclonal antibody drug is the only novel PD-1 monoclonal antibody that adopts immunoglobulin G1 (IgG1) subtype and performs Fc segment modification	2021
Zimberelimab	A fully human anti-PD-1 monoclonal antibody independently developed using the international advanced transgenic rat platform	2021

### 2.1 Checkpoint inhibitor

Immune checkpoints regulate the immune syste, where stimulatory immune checkpoint molecules promote the activation of T-cells and activate the immune response suppress the immune response of the body and prevent the development of autoimmunity (Liu and Xu 2020). Up to now, as many as 70 immune checkpoint inhibitors are in phase III and IV clinical trials; seven immune checkpoint inhibitors have been approved by FDA; and more than ten immune checkpoints have been discovered (Franzin et al., 2020).

Immune checkpoint molecules include CTLA-4, PD-1, PD-L1, which are the most widely researched, and other potentially novel immune checkpoint molecules that has not been used in the clinic, such as lymphocyte activationgene3 (LAG-3), T-cell immunoglobulin and immunoreceptor tyrosine-based inhibitory motif (ITIM) structural domain proteins, T-cell immunoglobulin mucin 3 (TIM-3), and T-cell activation inhibitor immunoglobulin variable region structural domain ISTA) (Mehta et al., 2019; Maruhashi et al., 2020; Fathi et al., 2021; Zhao et al., 2021).

CTLA-4, a transmembrane protein encoded by the CTLA-4 gene, is expressed in activated CD4^+^ and CD8^+^ T cells and binds to the ligands CD80 (B7-1) and CD86 (B7-2). It is also a T-cell surface receptor that acts as an immunosuppressive molecule and can participate in immunosuppressive signaling (Wei et al., 2020). CTLA-4 aborts the activated T-cell receptor (TCR) and mediates the suppressive function of regulatory T-cells (Treg). In addition, CTLA-4 mediates the binding of CD80/CD86 by dendritic cells and induces the expression of the tryptophan-degrading enzyme indoleamine 2, 3⁃dioxygenase, leading to the suppression of TCR (Anderson et al., 2016). CTLA-4 antibody reduces the inhibition of Treg and activates the TCR by binding to CTLA-4. In 2011, the FDA approved the first antibody drug working on immune checkpoint CTLA-4, ipilimumab (Kapadia et al., 2015), which is mainly used in treating melanoma and the prolonging of patient survival can reach to 1–2 years (Baas et al., 2021). Due to its early launch, limited monotherapy effect, and more adverse reactions, its combination therapy with PD-1 antibody have some prospects to amplify the tumor-suppressive effec.

In addition to CTLA-4, PD-1 is another common immunosuppressive molecule on the surface of T-cells, which are expressed on the surface of various tumor cells (Bai et al., 2020; Yang et al., 2022). PD-1, with two ligands: Is a member of the CD28 PD-L1 (also known as CD274 or B7⁃H1) and PD-L2 (also known as CD273 or B7⁃DC). PD-L1 is expressed by tumor cells binds to PD-1, blocking T-cell activation and cytokine production. PD-1/PD-L1 antibodies block the pathway by binding to PD-1/PD-L1, restoring immune killing function and enabling tumor immune escape (Zhang and Zheng 2020). Tumor cells express PD-L1 inhibit T-cell activation by binding to PD-1 on the surface of T cells, and PD-1/PD-L1 inhibitors close these immune checkpoints, and enhancing T-cell activity and killing tumor cells (Masarwy et al., 2021). The first PD-1 inhibitor, pembrolizumab, was approved in 2014 for the treatment of melanoma *versus* lung cancer (Yang et al., 2022). Then in 2016, the first PD-L1 inhibitor, atezolizumab, was approved for the treatment of bladder cancer (Liu and Xu 2020). Currently, clinical studies in solid tumors have found a conclution that overall survival with PD-1 antibodies better than with PD-L1 antibody therapy. However, there was no significant difference in the incidence of overall adverse events and immunotherapy-related adverse events (irAE) between the two groups. Results of head-to-head clinical trials comparing PD-1 and PD-L1 antibodies are not yet available (Duan et al., 2020).

### 2.2 Cellular immunotherapy

Cellular immunotherapy, also known as pericyte therapy, is also a type of immunotherapy (Reschke et al., 2021). Cellular immunotherapy is used to kill pathogens, cancer cells, and mutated cells in blood and tissues by extracting the body’s immune cells, proliferating them *in vitro*, increasing the targeting destruction, and infusing them back into the patient (Mizukoshi and Kaneko 2019). With the new research, developed, breakthroughs, and understanding of successive cellular immunotherapies in the global medical community, cellular therapy is gradually pushed to transform a “non-mainstream therapy” to an “adjunct to standard therapy” (Wang and Cao 2020). Chimeric Antigen Receptor T-cell therapy (CAR-T), which extracts T-cells from a patient’s own immune system, is cultured and modified *in vitro*, and equipping these T-cells with special molecules that enable them to recognize and attack specific cancer cells. The modified T-cells are injected back into the patient’s body and to destroy the immune response of cancer cells (Golubovskaya and Wu 2016). They are not restricted by human leukocyte antigens (HLA) because the antigen recognition sites of CAR-T cells are composed of monoclonal antibodies that specifically recognize tumor surface antigens (Mei et al., 2021). Two products have been approved by the FDA for clinical use (Al Hadidi et al., 2022). Another kind of pericyte therapy is named Engineered T-cell receptor therapy (TCR-T) which genetically modified immune effector cells are directly as anticancer therapy (Levy and Gros 2022). TCR-T therapy is performed by inducing the expression of modified T-cell receptors (TCRs) on the surface of T-cells, redirecting their endogenous cell killing activity to cancer cells. Currently, the TCR-T therapeutic agent CVT-TCR-01, which targets an intracellular cancerous testicular antigen called NY-ESO-1, has made great clinical progress (Zhang and Wang 2019). Tumor-infiltrating lymphocyte (TIL) therapy is a therapy based on tumor-specific T-cells, using infiltrating lymphocytes isolated from resected tumor tissue, expanding and culturing *in vitro*, and then transfusing back to patients to exert anti-tumor effects. It is a novel anti-tumor effector cell with the advantages of high efficiency, specificity, and low side effects. TIL therapy has been used clinically to treat primary or secondary tumors in the skin, kidney, lung, head and neck, liver, and ovarian sites (Stanton and Disis 2016; El Bairi et al., 2021). Natural killer (NK) cells in natural killer cell therapy are the third group of lymphocytes same as T-cell Band cells (Mao et al., 2021). NK cells in innate immune system and T lymphocytes (cytotoxic T lymphocytes) in postnatal immune system are two main types of cells used to attack malignant tumors. NK cells play an important role in natural hosts to the immune response of viruses and tumors. NK cell therapy requires the isolation from cord blood stored in cord blood banks to be genetic engineered to introduce CARs recognizing the surface targets of cancer cells before transfusion back to patients (Rafei et al., 2021).

### 2.3 Cytokine therapy

Cytokine therapy, also known as non-specific immunotherapy, is one of the first tools used in tumor immunotherapy. It mainly works through cytokines (e.g., IL-2, INF), immune adjuvants, and short peptides to enhance the body’s immune response. Substances used to regulate immunity are often referred as immune system modulators. The initial cytokine drugs approved by the FDA were IFNα-2a (Roferon-A) and IFNα-2b (Intron-A). Subsequently, high doses of IL-2 (Proleukin) were approved for the clinical treatment of metastatic melanoma and renal cancer (Khan et al., 2021). In addition, the immune-adjuvant class is not antigenic *per se* but enhances the stimulation of the body’s immune system by antigen. Immune restorer-type modulators can restore the suppressed immune reaction into normal levels. It treats cancer through the overall upregulation of the body’s immune function from different aspects. Immunomodulators cannot identify immune cells from enemy cells, and may “accidentally” harm normal cells and lead to certain adverse effects. Moreover, there appears to be a dose response for IL-2, as it appears that the most effective dose is also associated with significant toxicity. The most important cause of toxicity is vascular leakage syndrome, which manifests as fluid loss into the interstitial space as a result of increased vascular permeability. Other effects include thrombocytopenia, elevated hepatic serum transaminases, hepatocyte necrosis, hypoalbuminemia, tissue and peripheral eosinophilia, and pre-renal azotemia (Mier et al., 1988). It was shown that only very high intravenous doses lead to adequate clinical responses, but administration leads to severe, poorly tolerated, reversible but often life-threatening multisystem toxicity that may cause life-threatening side effects in patients (Rosenberg et al., 1985). IL-2 therapy, although one of the oldest successes of cancer immunotherapy to date, is still not widely implemented due to the high toxicity associated with it. To present, one drug (Ceplene, histamine dichloride) has successfully entered regulatory and clinical thresholds as a drug to reduce IL-2 toxicity and enhance efficacy (Hornyak et al., 2005). Thus, a regulatory pathway has been established for the development of such a drug. Therefore, cytokine therapy is often used in combination with other therapies for treatment or with adjuvants in order to reduce the associated toxicity (Passalacqua et al., 2014).

### 2.4 Problems in immunotherapy

Tumor immunotherapy mainly relies on regulating or activating the host’s immune system to inhibit or kill tumors, which has the advantages of low toxicity and high efficiency. However, challenges in clinical tumor immunotherapy are still need to be solved. (Routy et al., 2018). First, the similarity between tumor cells and normal cells leads to difficulties for human immune system to distinguish them correctly (Sau et al., 2018). For example, CAR-T therapies can attack tumor cells and attack other normal cells as well, which is also known as “off-target effect” (Song et al., 2015). Secondly, solid tumors have an immunosuppressive microenvironment, preventing some injected immune cells or cytokines from reaching the tumor site successfully, and leading to effective in hematologic tumors in immunotherapy. However, the problem of attacking solid tumors cannot be solved (Jiang et al., 2017). With the development of materials science and nanotechnology, nanomedicines have the following advantages that are expected to provide new ideas for improving the effectiveness of immunotherapy: First, nanomedicines can enhance drug accumulation at the tumor site due to their unique high permeation and long retention (EPR) effect, thus therapeutic effect is improved (Li et al., 2011). Second, some nanomaterials have special properties, including temperature-sensitive properties (Li et al., 2011) and pH-sensitive properties Through nanotechnology, modified drugs can achieve dual targeting actively and passively, reduce drug concentration and improve therapeutic tolerance while enhancing targeting (Li et al., 2011; Zhu et al., 2017). Third, by optimizing the morphological characteristics and surface properties of nanomedicines (Xu et al., 2012), the half-life in the circulation of the reticuloendothelial system can be prolonged to prevent its rapid clearance by immune syst hematological malignancies em (Li et al., 2012). Therefore, the use of nanomedicines can overcome some shortcomings of immunotherapy and enhance the effect of tumor immunotherapy and provide more effective treatments for patients through further development of nano immunotherapy.

### 3 Nanomedicine

Nanomedicines, as an emerging technology, have great efficiency in tumors, cellular oxidative damage, and many other diseases because of features including small particle size and stable surface function (Golubovskaya and Wu 2016; Wang and Cao 2020). One of the most important applications of nanoparticles is anti-tumor, improving the effectiveness and persistence of immunotherapy. The main methods include enhancing tumor drug enrichment, reducing drug resistance in the form of modified drugs and increasing the half-life of the modified drugs (Figueiras et al., 2022).

### 3.1 Types and characteristics of nanomedicines

Nanomedicines include polymeric micelles, liposomes, nanoemulsions, dendritic macromolecules and inorganic nanoparticles. Their main characteristics are shown in Table 2. As the main type of nanomedicines, polymeric micelle, has advantages of core-shell structure, high drug-carrying capacity, targeting ability, biodegradability, and stability (Abdel-Hafez et al., 2018). However, polymeric micelles have some shortcomings, such as toxicity and instability in the blood (Wang et al., 2014). Therefore, polymeric micelles should be intervened before being used in such conditions. Intervening methods include polyethylene glycolization and chain length improvement (Mao et al., 2017). As carriers, liposomes has the advantages of high biosafety, strong activity, rapid release, high bioavailability, high efficiency, rapid detection speed and long-term preservation in a circulating environment (Cheng et al., 2018). However, liposomes have disadvantages such as difficult to overcome biological barriers, for example, the blood-brain barrier and the blood-tumor barrier (Satta et al., 2022). Nanoemulsions can improve encapsulation efficiency, improve stability, reduce adverse effects, improve safety and enhance solubility (Peer and Margalit 2004; Cheng et al., 2022). However, its limitations, contain higher costs and short period for storage (Lakshmanan et al., 2021). Dendritic macromolecular nanomaterial has features including reducing dose of the drug, increasing drug effeciency, modulating biodistribution, reducing side effects, improving delivery of targeted drug detecting rapidly by fluorescence test and improving loading capacity (Sabjan et al., 2020; Mohamad et al., 2021). However, it faces issues of equipment and theory shortage, and high expansion, etc (Delyanee et al., 2021). Therefore, at present, the development of dendritic macromolecules mainly focus on generalization and economization. With unique magnetic and optical properties, inorganic nanoparticles can control nano-structural properties for effective transport, overcome biological barriers at the cellular and tissue levels, and replenish the body with essential elements and substances (Bober et al., 2022). In addition, it has the advantages of mucosal penetration, skin penetration and systemic circulation.

**TABLE 2:** Types and main characteristics of nanomedicines.

The types of nano drug	Advantages	Disadvantages	References
Polymeric micelle	Core-shell structure, high drug-carrying capacity, targeting, biodegradable and stable	Toxic, unstable in blood, requires early intervention by means of polyethylene glycolization and increased chain length	Fang et al. (2014); Overchuk et al. (2020); Liu et al. (2021b)
Liposome	High biosafety, high activity, fast release, high bioavailability, and in a circulating environment	Difficulty in overcoming biological barriers	Xue et al. (2019); Dai et al. (2020); Lu et al. (2020); Wei et al. (2020); Zhang et al. (2021)
Nanoemulsion	High encapsulation efficiency, high stability, low adverse reactions, enhanced safety, and enhanced solubility	Higher cost, not easy to store	Bai et al. (2020); Liu and Xu (2020); Yang et al. (2022)
Dendritic macromolecules	Reducing necessary drug dose, increasing effectiveness, improving drug delivery, and allowing for rapid detection by fluorometric testing	Less theoretical research and expensive equipment	Dong et al. (2002); Buchbinder and Desai (2016); Sul et al. (2016); Rowshanravan et al. (2018); Liu et al. (2021a)
Inorganic materials-based nanoparticle	Magnetic and optical properties that provide control of nanostructural properties for effective drug transport, allowing mucosal penetration, skin penetration and systemic circulation	Primarily used for *in vivo* imaging, drug delivery studies are still in the experimental stage, with long-term potential toxicity and low clearance issues	Korman et al. (2006); Ning et al. (2017)

Currently, researchers constructed d-α-tocopherol polyethylene glycol 1000 succinate (TPGS)-modified carboxymethyl chitosan rhodopsin (TCR) polymeric micelles (PMs) for the oral administration of paclitaxel (PTX) (Wang et al., 2022). The results showed that TCR PMs with a drug loading of 47.52 ± 1.65% can significantly improve the intestinal absorption and oral bioavailability of paclitaxel. Furthermore, nanomedicines exerted the effects of altering allergic phenotypes, prolonging half-life, overcoming biological barriers at cellular and tissue levels, improving antitumor effects and reducing side effects (Mao et al., 2017), through the development of nanoemulsion vaccines (O’Konek et al., 2020), dendritic macromolecular nanoparticles (NP-TPGS-SFB) (Li Z et al., 2020), inorganic nanoparticles (Xie et al., 2019), cisplatin (CDDP) and curcumin (CUR) co-loaded liposomes (CDDP and CUR-Lip) (Cheng et al., 2018) and novel bee toxin nanoliposomes.

Based on the properties of each material, it is known that polymeric micelles can be used for oncology treatments, drugs with high targeting requirements, etc.; nanoliposomes are recommended for transporting small polar drugs such as paclitaxel and artemisinin; nanoemulsions can be used for drugs with poor solubility and encapsulating capacity, like ophthalmic drugs, oral agents and injectables; dendritic macromolecules can be used when there is a need for large volumes and rapid detection; apheresis nanoparticles can be used when metal trace elements are shortening and biological barriers cannot be overcome.

### 3.2 Drug delivery strategies

**FIGURE 1:**
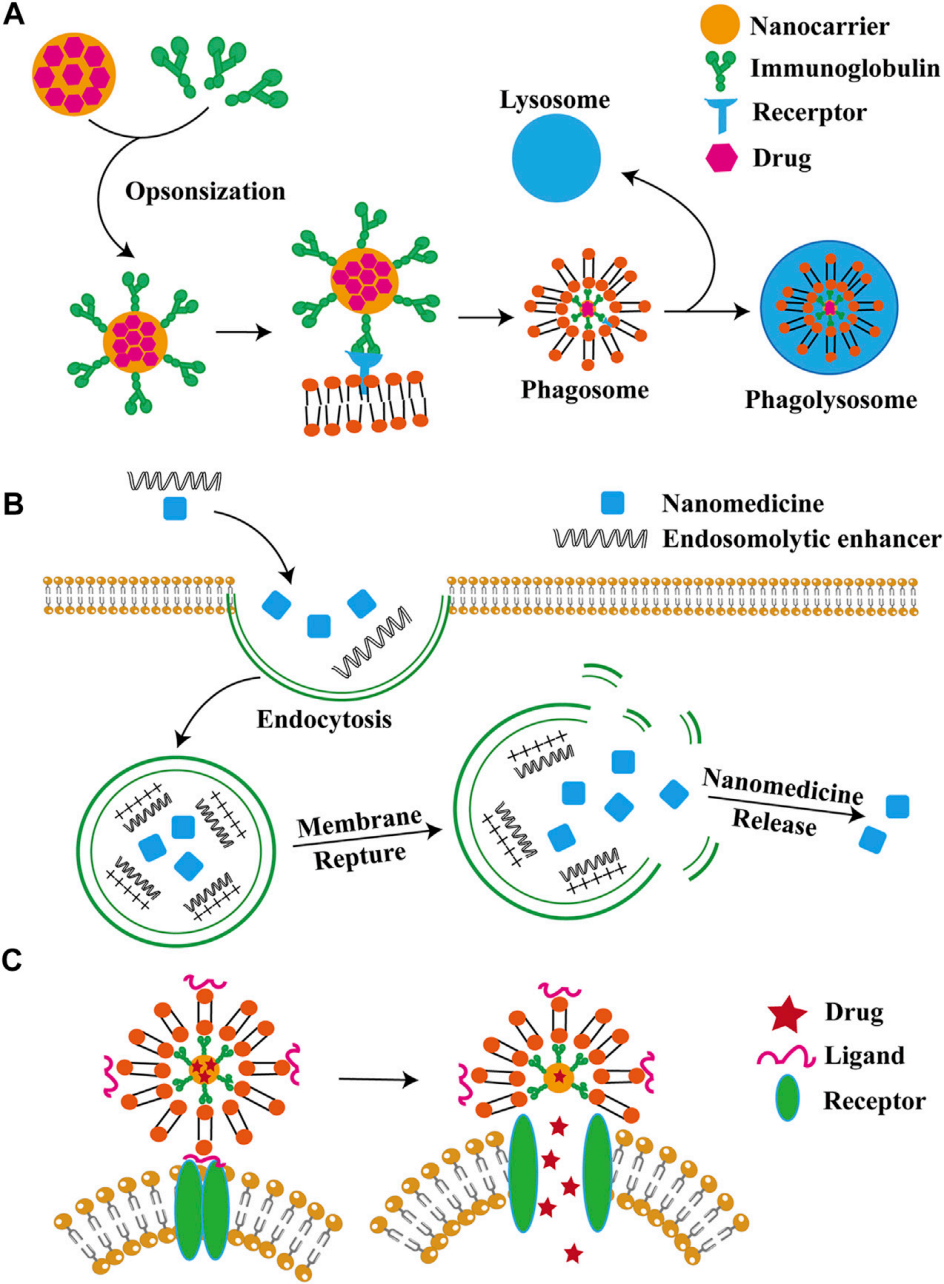
Major nanoparticle delivery strategies. Drug delivery of nanoparticles is mainly through cellular uptake, intracellular solute delivery and intracellular direct delivery, as shown in (A–C), respectively.

Drug delivery of nanoparticles mainly contains three ways: cellular uptake, cytosolic delivery by endosome escape and direct intracellular delivery, as detailed in Figure 1A–C.

#### 3.2.1 Cellular absorbing mechanisms

Nanoparticles (NPs) can enter into cells by endocytosis. In thisprocess, cell membrane engulfs NPs and forms a separate vesicle in the cell. The mechanisms of endocytosis include phagocytosis, latticin- and niche protein-mediated endocytosis, and latticin- and niche protein-independent pathways (Lee et al., 2021). Phagocytosis occurs in immune cells, such as dendritic cells, T cells, B cells, neutrophils and macrophages, which work as means of removal of foreign bodies (Gordon 2016).

#### 3.2.2 Endosomal escape

Nanoparticles with enhanced endosomal escape ability are the best choice for bio-delivery. Commonly used nanoparticle that are commonly used for endocytosis and biological agents’ delivery includes liposomal and lipid-based NPs, polymer micelles and polymer NPs, lipid-polymer hybrid NPs, nanocrystals, and inorganic NPs such as gold NPs, magnetic NPs, graphene, quantum dots, and silicon NPs. The mechanism of intranuclear body escape involves instability of the intranuclear body membrane, which can be solved by increasing the interaction between the intranuclear body membrane and intranuclear solvents such as pH-sensitive solvation compounds or polymers (Wang et al., 2016). Currently, the main strategies for endosome escape include endosomes lysis with intranuclear enhancers; endosome membrane pore formation; effect of high pH buffers mediating the proton sponge; fusion agents interference of forming membrane fusion; and the application of photosensitizers causing photochemical destruction of endosome membranes (Lorenz et al., 2014).

#### 3.2.3 Direct intracellular delivery

Various methods have been developed to enhance intranuclear body escape, including delivering cytoplasmic directly bypassing intranuclear body wrapping and delivering organisms to the cytoplasm directly. The solutions contain fossa-mediated pathways, contact release, and intermembrane transfer, membrane fusion, direct intracellular translocation, membrane rupture, and microinjection (Liu et al., 2014).

Although nanocarriers is developing rapidly, many challenges are still to be addressed. The intracellular drug delivery and efficacy of nanocarriers are mainly controlled by the mode of internalization and intracellular transport. Endocytosis of NPs is a major pathway, but intranuclear endosomal encapsulation leads to a lower possibility of cytoplasmic delivery. Materials for inducing intranuclear endosome escape can be limited by toxicity (Zhang et al., 2018). To solve this problem, choosing alternative drug delivery or reducing the potential toxicity of nanoparticles. Improving poor intracytoplasmic drug delivery can be achieved as well. Direct intracytoplasmic delivery is a promising intracellular delivery for biologics because its rate of delivering into cytoplasm can almost achieve 100%, However, more safety issues needed to be addressed (Schmid et al., 2017). Safe and scalable NPs is still a focus of translational biology.

### 3.3 Nanoparticles and coupling substances

Nanoparticles and coupling compounds are widely used in the following six areas.

#### 3.3.1 Vascular receptor-mediated drug in immune cells

Immune cells play a crucial role in the immune response process. However, the continuous activation of the immune system may disrupt immune homeostasis and lead to inflammatory diseases. Targeted immunomodulators may be useful in the treatment of such diseases and their complications. However, the disadvantages of immunomodulators include high immune-mediated toxicity, low solubility, and) loss of biological activity after long-term circulation (Ko et al., 2022). At this stage, nanocarriers have become a very promising tool to overcome these barriers due to their unique properties such as continuous circulation, ideal biodistribution, and preferred pharmacokinetic and pharmacodynamic properties. It has become one of the irreplaceable materials in the delivery of immunomodulatory drugs and vaccine adjuvants.

#### 3.3.2 Nanoparticles for mRNA cancer vaccines

Among the many cancer vaccines, mRNA is becoming more prevalent for the following three reasons (Miao et al., 2021). First, mRNA can encode multiple antigens simultaneously, or contain whole proteins with MHCI and MHCII binding epitopes, which can promote humoral and cellular adaptive immune responses and enhance anti-tumor immunity. Second, compared with DNA vaccines, mRNA vaccines has features of non-integrated highly degraded, and lack of insertional mutagenic potential. In contrast to protein or cell-mediated vaccines, it does not contain cellular and pathogenic viral components, meaning no potential for infection. Most mRNA vaccines tested in ongoing clinical trials are generally well tolerated and cases show a reactions at injected part. Finally, production of mRNA cancer vaccines is rapid and scalable. In addition, nanoparticles in mRNA vaccines has the following advantages: retaining activity of the encapsulated adjuvant and significantly improving the efficiency of mRNA transfection. It induces an effective adaptive immune response by increasing the expansion and the infiltration into the lesion *in vivo* of specific T cells (Islam et al., 2021). Effective delivery of the mRNA vaccine will be critical to success clinical application. Studies have shown that lipid nanoparticles can be synthesized easily in a scalable manner to protect mRNA from degradation, facilitate endosome release, modify the surface of ligands to achieve desired cell types targeting, and co-deliver adjuvants as needed (Miao et al., 2021).

#### 3.3.3 Ionizable lipid nanoparticles for *in vivo* mRNA delivery

Gene therapy, including mRNA delivery *in vivo*, has great potential for treating almost any disease, lipid nanoparticles are increasingly well-known as an excellent lipophilic gene therapy delivery vehicle. The study used bounded mRNA lipid nanoparticles as a carrier for targeted delivery because of two inspirations: the principle of absorption of apolipoprotein E (ApoE) to the surface of lipid nanoparticles, and the principle of achieving specific hepatocyte targeting. Studies have shown that lipid nanoparticle particles have better stability, biodistribution, and targeting capacity, making this technique suitable to be developed, having the potential of an extrahepatic targeting to develop a novel gene therapy (Francia et al., 2020). Libraries of ionizable lipid nanoparticles are designed to encapsulate mRNA to prevent its degradation, and mediate intracellular delivery. However, these ionizable lipid nanoparticles are typically characterized and screened *in vitro* environments, which may not fully replicate the biological barriers encountered. To address this problem, Guimaraes et al. designed an *in vivo* platform for accelerating mRNA delivery screening. This platform includes an engineered LNP library with functions of encapsulating and custom-designing barcoded mRNA (b-mRNA). These b-mRNAs are structurally and functionally similar to conventional mRNAs and contain barcodes that allow them to be quantified by deep sequencing (Guimaraes et al., 2019). The platform enables direct barcoding and subsequent quantification of functional mRNA, which can accelerate the *in vivo* screening and designing of ionizable lipid nanoparticles for mRNA therapeutic applications, such as CRISPR/Cas9 gene editing, mRNA vaccination and other mRNA-based regenerative medicine and protein replacement therapies. Hald et al. suggested that lipid nanoparticles play an important role in the mRNA vaccine against COVID-19 (Hald Albertsen et al., 2022). A growing number of data confirms that the scope of lipid nanoparticle-based gene therapy and vaccines treatment will be determined by the kind of lipid components are added and combined when used as carriers.

#### 3.3.4 Biologically inspired molecular coupled subunit vaccines

The development of effective vaccines is essential to prevent potential epidemics. However, there is a standard for rapid design and development of effective vaccines. Inspired by the natural budding process of invading a host cell to replicate, and fracturing cell membrane, a similar strategy was applied to achieve virus-mimicking nano vacuoles. This is achieved by molecularly coupled subunit vaccines, a strategy that loading genetically engineered viral antigens onto mammalian cell membranes to produce antigen-loaded vesicles, and then being optimized for size and stability by surfactants (Mi et al., 2016).

#### 3.3.5 Matrix-binding checkpoint inhibitor couples

CD40 is an immune co-stimulatory receptor expressed by antigen-presenting cells. Agonistic anti-CD40 antibodies have shown significant antitumor effects but may also cause serious adverse events such as hepatotoxicity. Ishihara et al. designed a variant to enhance the effect of partial administration of anti-CD40 through extracellular matrix of a super affinity peptide derived from placental growth factor-2. It proves the significant therapeutic advantage of cancer immunotherapy, from engineering matrix-binding structural domains to agonistic anti-CD40 antibodies injections (Ishihara et al., 2018).

### 3.4 Current situation and problems

As an emerging drug delivery system, nanomaterials have advantages and disadvantages. At this stage, the current status and problems of common nanomaterials (targeted ligand nanomaterials, nanomedicine delivery systems, size and surface modification of nanomedicine formulations, etc*.*) are shown below (Table 3).

**TABLE 3:** Status and problems of nanoparticles.

Materials (paths)	Situataions/questions	Perspectives/mearsures	References
Ligand-targeted particulate nanomedicines	1) It was proved safe and effective in preclinical models; 2) for the treatment of cancer; 3) Some contributions have not been clearly demonstrated; 4) it does not lead to localization in the target tissue, but provides the benefits of target cell internalization and target tissue retention after reaching the target site	1) Drug delivery that cannot effectively be passed through the cell membrane; 2) treatment of drug-resistant tumors; 3) targeting of tumor blood supply; 4) production of targeted vaccines; 5) nanomedicines that can cross the blood-brain barrier	Wang et al. (2020)
Nanomedicine delivery system	1) Improve drug safety and efficacy; 2) it could enter the intracellular domain of diseased cells	1) Focus on the particle size of nanomedicines, which are the core and key quality attributes; 2) The process used to characterize new and complex nuclear power sources should be compatible with references materials	Jaunalksne et al. (2020)
Size and surface modification of nanopharmaceuticals	1) Mastered the method of measuring various solid nanomedicines; 2) evaluating the size of polydisperse nanomedicines; 3) challenges in the development of methods for measuring non-spherical nanomedicine	1) Although surface modification methods for analyzing nuclear power sources have been established, the feasibility of their application to nuclear power sources is unclear; 2) Determination of lack of available references materials and difficulty in selecting suitable materials for surface characterization of modified nanomedicine; 3) Research and development of cancer drugs and gene therapies for cells, tissues and organs are underway; 4) Next-generation nanomedicine should focus on the practice and standardization of nanomedicine measurement methods to design surface modifications and ensure their quality	Ramselyte et al. (2021)
Eudragit-Chitosan Coated	Advantages of temozolomide loaded Eudragit-chitosan coated selenium nanoparticles:1) slow and pH sensitive release kinetics; 2) compared with free curcumin, it showed better C6 cell uptake performance. Compared with free temozolomide and the control group, the coated nanoparticles loaded with temozolomide showed higher cytotoxicity and apoptosis	Nanoparticles and chitosan coating composite can reduce the drug resistance and side effects of some drugs, the future can be used for poor efficacy or drug-resistant drugs	Zeng et al. (2021)
Nano-delivery system	1) Limit drug toxicity; 2) potential of tumor localization; 3) nanocarriers can be used to target tumor-specific receptors, tumor antigens and tumor vessels with high affinity and precision; 4) reduces toxicity other parts of the body and protect cytotoxic drugs from degradation; 5) increased half-life of cytotoxic drugs; 6) reduce renal clearance	1) New means of anticancer drugs; 2) various delivery routes from oral to transdermal; 3) drug efficacy, pharmacokinetics and biodistribution superior to traditional drug solutions	Song et al. (2015); Santoni et al. (2018); Sau et al. (2018); Zeng et al, (2021)
Stereostable liposomes containing cyclic peptides	The activation of hepatic stellate cells contributes to the development of liver fibrosis. In order to activate hepatic stellate cells, a stereostable liposome containing cyclic peptide was established. The cyclic peptide has a high affinity for αvβ2 to achieve aHSC specific delivery	1) Cyclic peptide-guided liposomes are preferentially internalized by activated hepatic stellate cells *in vitro* and *in vivo*, and depletion of excess free cyclic peptides eliminates internalization; 2) The nanoparticle system shows great prospects for delivering therapeutic agents to activated hepatic stellate cells to treat liver fibrosis	Li et al. (2011)
Nanomedicine Co.—delivery system of natural active ingredients and chemotherapy drugs	Drug combination therapy and co-delivery systems of natural active ingredients and chemotherapy drugs offer great advantages	1) Natural products are innovative sources of anticancer drugs and natural active ingredients have effects on cellular processes and signaling pathways; 2) effective encapsulation of natural active ingredients and chemotherapeutic drugs; 3) this puts higher demands on the complex structure of the carrier; 4) Continuous and precise drug release *in vivo*	Li, et al. (2011)
Nanomedicine delivery system for targeted therapy of liver cirrhosis	1) CeO_2_NPs; 2) silver nanoparticle; 3) dexamethasone-liposomes; 4) cationic liposome microRNA; 5) Vitamin A coupled liposomes; 6) berberine bovine serum albumin nanoparticles	Many problems need further study, such as some hepatotoxicity of silicon nanoparticles. With the latest development of safe and efficient nano drug delivery systems, it could serve as a future solution for the treatment of various liver diseases	Li et al. (2011)
Regulatory mechanism of nano—delivery system on breast cancer through inflammatory microenvironment	1) High bioavailability; 2) Reduction of dosage 3) Eliminate target side effects; 4) Reduced tumor angiogenesis; 5) No systemic toxicity	Prevent and control breast cancer and other cancers through nanomedicine delivery systems. More effective cell/tissue models, such as 3D cell sphere culture models, are needed to verify the role of the drug delivery system	Li et al. (2012); Zhu et al. (2017)
Poly (lactic-co-glycolide) (PLGA) nanoparticles	1) PLGA system can be used for the delivery of proteins, peptides, vaccines, genes, antigens, growth factors and other substances; 2) the encapsulation efficiency and release rate of nano/particle-mediated drug delivery device can be optimized to improve its therapeutic effect; 3) the system has good biocompatibility and degradability	At present, the system can be used to treat Ehrlich ascites tumor. The combination of various compounds (mainly PEG, CS, PVA and iron oxide) into PLA-based nanoparticles and microparticles provides the possibility to expand the applicability of these materials	Abdel-Hafez et al. (2018); Li W et al. (2020); Yu and De Geest (2020); Figueiras, Domingues et al. (2022)
Nanoparticles and coronavirus (COVID-19 as an example)	1) Newer therapeutic targets can be identified and targeted using surface-functionalized nanoparticles; 2) provide the basis for the production of effective nano-vaccines; 3) gold, silver, silver sulfide, titanium oxide, zirconium, graphene and some biopolymer compounds have become the most suitable nanomaterials	1) Development of broad-spectrum universal nanovaccine or therapeutic vaccine; 2) Microfluidics has rapid detection and portability in the future; 3) The key to prevention is that small objects that may bind to the virus must be designed and made available in large quantities so that the virus can bind to them rather than to cell membranes; 4) It is urgent to develop reliable artificial intelligence and deep learning models	Wang et al. (2014); Mao et al. (2017); Cheng et al. (2018); Satta et al. (2022)

## 4 Application of nanomedicine in antitumor immunotherapy

### 4.1 Nanomedicines in immune checkpoint blockade (ICB) therapy

The development of immune antibodies has revolutionized cancer immunotherapy. Studies have demonstrated that nanomedicines can be targeted and retained at tumor sites with less adverse effects (Kulkarni et al., 2016). Nanomedicines derived from anti-CTLA-4 and anti-PD-L1 antibodies can inhibit tumor growth and reduce adverse effects (Kulkarni et al., 2016). Targeted delivery of ICB antibodies to residual tumor sites with platelet nanomedicines can inhibit tumor recurrence and treat tumor recurrence and treats metastasis with high clinical relevance (Lamla and Erdmann 2004; Lee et al., 2012). Wilson’s team deliver intracellularly interferon gene stimulator (STING) agonists by damaging polymers of endosomes, which is a natural form and difficult to cross the cell membrane. Treatment with these polymeric vesicles significantly improves the efficacy of anticancer immunity and checkpoint blocking therapies (Li et al., 2016).

### 4.2 Application of nanomedicines in modulate immunosuppressive cytokines modulation

Soluble cytokines is one of the essential method for tumor immunotherapy. The cytokine TGF-β can decrease the number and activity of NK cells and tumor-infiltrating T lymphocytes (CTL), also affect the number of regulatory T-cells. Irvine’s group investigated the immunosuppressive effects of small molecule inhibitor TGF-β by T-cell-targeted delivery a (SB525334) (Derynck, Turley et al.). Fahmy and others co-loaded the nanoparticles with TGF-β inhibitors and interleukin 2, attenuating immunosuppression and enhancing T-cell proliferation. Huang et al. (Park et al., 2012) developed a nano-formulation TGF-β siRNA that achieves approximately 50% knockdown of TGF-β expression in tumors and synergizes with cancer vaccination. For example, it shows more on cytotoxic T-cell infiltration and significantly inhibits tumor growth (Xu et al., 2014). In addition, liposomes that modified with ligands targeting T-cell internalized receptor (CD90) or non-internalized receptor (CD45), are tested their performance, revealing that both two liposome agents can induce high expression of CTL in tumors. Furthermore, lower tumor suppression was observed with anti-CD45 liposomes than with anti-CD90 liposomes. This observation was attributed to the fact that the receptor CD45 was normally expressed by nuclear hematopoietic cells (Simpson et al., 2015). It was also shown that peripheral blood B cells, dendritic cells and macrophages did not ideally internalize anti-CD45 liposomes (Lu et al., 2016).

## 5 Chimeric antigen receptor T-cell immunotherapy and applications

### 5.1 Chimeric antigen receptor T-cell immunotherapy

**FIGURE 2:**
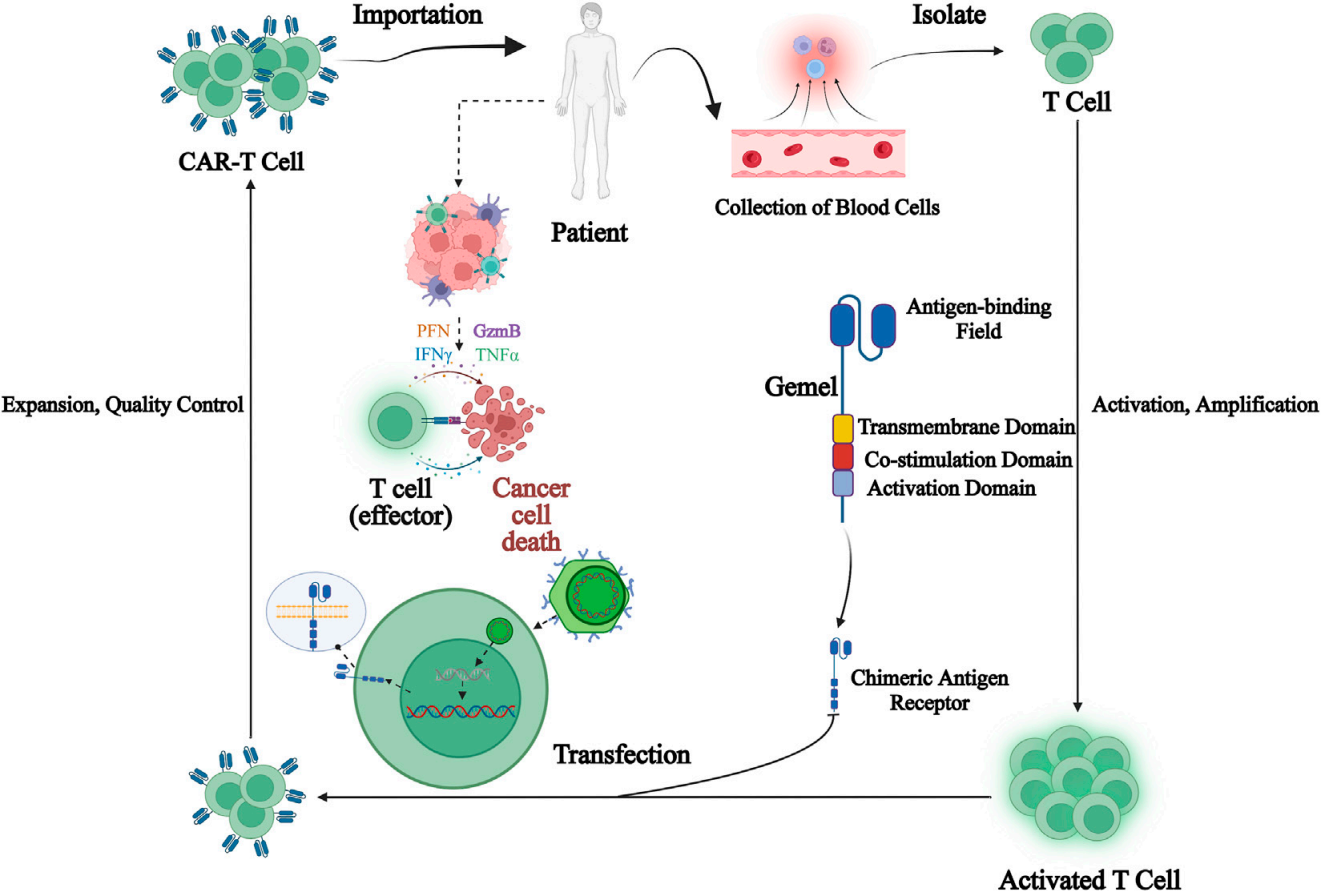
Chimeric antigen receptor T (CAR-T) cell immunotherapy process. CAR-T therapy is performed by collecting peripheral blood from patients and isolating lymphocytes from it, introducing CAR genes into T-cells through genetic engineering techniques to express specific receptors to bind specific tumor-associated antigens, amplifying them *in vitro* and then transfusing them back to patients, and after specific binding of CAR-T-cells to tumor-associated antigens *in vivo*, activating T-cells through signal transduction, promoting T-cell proliferation and releasing cytokines to exert anti-tumor effects.

Since its introduction, chimeric antigen receptor T-cell immunotherapy (CAR-T) has shown greater clinical advantages and potential. CAR-T therapy is worked by collecting peripheral blood from patients and isolating lymphocytes, introducing CAR genes into T-cells through genetic engineering techniques to express specific receptors to bind specific tumor-associated antigens. After being a amplified *in vitro*, they are transfused back to patients specifically binding of CAR-T cells to tumor-associated antigens *in vivo*, activating T-cells through signal transduction, promoting T-cell proliferation and releasing cytokines to exert anti-tumor effects (Gauthier and Yakoub-Agha 2017). Typically, a CAR is a fusion protein consisting of a combination of an extracellular antibody variable region used to recognize a tumor antigen site and an intracellular signal transduction module from a T-cell. Single-chain antibody light and heavy chain variable regions scFv fragments in the extracellular are responsible for recognizing and binding to tumor antigen targets. Hinges in the transmembrane region provide flexibility in antibody recognition. CD28 and CD3ζ in the intracellular signal transduction region are responsible for delivering activation and proliferation signals to the cell (Kosti et al., 2018). After being activated, CAR-T cells secrete a large number of cytokines activating themselves as well as other immune cells rapidly and induce apoptosis of target cells (Figure 2).

**FIGURE 3:**
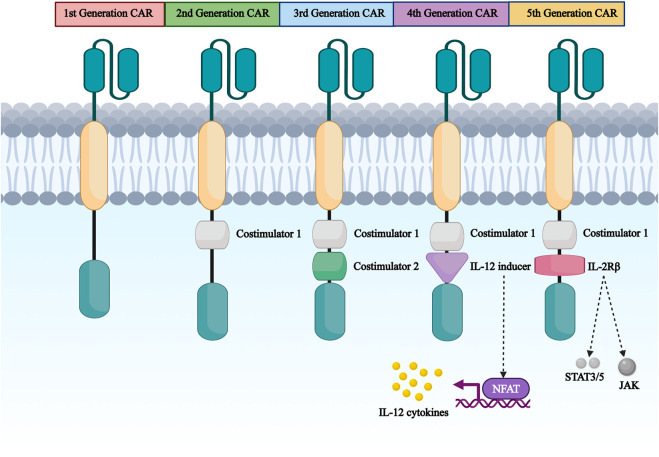
The first generation to the fifth generation CAR structure diagram. CAR are divided into five generations according to their intracellular domains. The first generation has a single activating structural domain, mainly the cytoplasmic structural domain of CD3ζ.The second generation has CD3ζ plus a co-stimulatory structural domain obtained from co-stimulatory molecules such as 4-1BB or CD28 attached to the activating structural domain. The third to fifth generation are based on the first or second generation of design.

CARs are classified into five generations based on intracellular structural domains: The first generation has a single activating structural domain, mainly CD3ζ, and some research studied on the gamma chain of the Fc receptor. The second generation added a co-stimulatory structural domain obtaining from co-stimulatory molecules such as 4-1BB or CD28 attaching to activate structural domain (CD3ζ/γ chain of the Fc receptor) to enhance the cell proliferation and cytotoxic capacity of CAR-T cells (Acuto and Michel 2003). The third generation is similar to the second generation but has multiple structural domains co-stimulated with CD3ζ, such as 4-1BB and CD28, CD134 and CD137 (Fang et al., 2014). The fourth-generation CARs, known as T-cells retargeted cytokine-mediated killings (TRUCKs), were designed to release the transgenic cytokine IL-12 during CAR signaling in tumor tissue to overcome TME immunosuppression and achieve robust therapeutic outcomes (Li W et al., 2020). IL-12 is responsible for the induction of IFN-γ, perforin and granzyme in T-cells and inhibits Treg proliferation (Yu and De Geest 2020). Other cytokines mentioned in the fourth generation are IL-15 and IL-18 (Xue et al., 2019). IL-15is γ-chain segment and it has important properties of expansion and survival for T-cells (Cao et al., 2009). In addition, IL-18 CAR T-cell treatment of large pancreatic and lung tumors showed changes in the immune cell landscape; significant increases in macrophages (CD 206 M1) and NKs (NKG2D+) were observed Apart from this, a decrease in Tregs such as M2 macrophage-suppressive CD 103 + DCs, indicates the ability of “IL-18 TRUCKs” to sensitize large tumor lesions for effective immune destruction (Hurton et al., 2016). Fifth-generation CAR-T therapies, based on the second-generation are being explored. It contains a truncated cytoplasmic receptor (IL-12), a β-chain structural domain (IL-2Rβ truncated intracellular interleukin two β-chain receptor) as well as the transcription factor STAT3/5 binding motif (Klebanoff et al., 2004) (Figure 3).

### 5.2 Application of nanomedicines in CAR-T therapy

Traditional cancer treatments often cause damage to normal cells in the body, or residual malignant tumor cells flow into the bloodstream through the wounded area, making poor prognosis. Moreover, single immunotherapy modalities have limitations in their efficacy when treating solid tumors. Based on this, the use of combined nanoparticles for cancer immunotherapy can achieve synergistic effects. The current limitations of CAR-T immunotherapy in clinical tumor immunotherapy include the following: stimulating T-cell expansion and persistence; improving T-cell trafficking; stimulating intrinsic T-cell activity; resetting immunosuppressive cells and vascular microenvironment; methods to monitor the therapeutic efficacy of CAR-T cell therapies, etc.

#### 5.2.1 Nanomedicines stimulates T-cell expansion and persistence

The complex preparation procedures and severe toxicities are two major obstacles facing the wide use of chimeric antigen receptor-modified T (CAR-T) cells in clinical cancer immunotherapy. The nanotechnology-based T-cell temporary CAR modification may be a potential approach to solve these problems and make the CAR-T cell-based tumor therapy feasible and broadly applicable. To solve this problem, Judith Guasch (Guasch et al., 2018) propose to activate primary human T-cells using a combination of nanostructured surfaces functionalized with the stimulating anti-CD3 antibody and the peptidic sequence arginine-glycine-aspartic acid, as well as costimulatory agents (anti-CD28 antibody and a cocktail of phorbol 12-myristate 13-acetate, ionomycin, and protein transport inhibitors). This method that combines nanotechnology with cell biology procedures to efficiently produce T-cells in the laboratory, challenging the current state-of-the-art expansion methodologies. Qianru Yu’s study (Yu et al., 2020) showed that a series of plasmid DNA-loaded self-assembled nanoparticles (pDNA@SNPsx/y) prepared from adamantane-grafted polyamidoamine (Ad-PAMAM) dendrimers of different generations (G1 or G5) and cyclodextrin-grafted branched polyethylenimine (CD-PEI) of different molecular weights (800, 2000, or 25,000 Da) were characterized and evaluated. The detailed physicochemical properties, cellular interaction, and cytotoxicity of selected pDNA@SNPG1/800 were systematically investigated. Finally, the response of EGFRvIII CAR-positive Jurkat T-cell to target tumor cell was evaluated. The results indicated that pEGFRvIII-CAR@SNPG1/800 can effectively achieve T-cell transient CAR modification, thereby demonstrating considerable potential in CAR-T cancer therapy. Subsequently, scientists have developed hydrogels (Guasch et al., 2017, Pérez Del Río et al., 2020), 3D hydrogels (Santos et al., 2022), nanocapsules (Thiramanas et al., 2020), nanoparticles (Yu, Zhang et al., 2020), and other nanoformulations to reduce cytotoxicity and achieve the goal of stimulating T-cell expansion and persistence.

#### 5.2.2 Nanomedicines enhance the effectiveness of CAR-T cells

Chimeric antigen receptor (CAR)-modified T-cell therapy has the potential to improve the overall survival of patients with malignancies by enhancing the effectiveness of CAR T-cells. Precisely predicting the effectiveness of various CAR T-cells represents one of today’s key unsolved problems in immunotherapy. Qin Liu (Liu et al., 2020) encapsulated interferon-α2b (IFN-α2b) into microporous hydrogels as an enhancement factor and utilized low-dose irradiation (LDI) as a tumoral attractor of T-cells. The results show that hydrogels kept the activity of IFN-α2b and stably release of IFN-α2b to stimulate T-cells for a long time. At the same time, low-dose radiation recruits T-cells into tumors. This innovative integration mode of IFN-α2b-loaded hydrogels and radiotherapy offers a potent strategy to improve the therapeutic outcome of T-cell therapy. Xiong W and colleagues (Xiong et al., 2018) predict the effectiveness of CAR-modified cells by evaluating the quality of the CAR-mediated immunological synapse (IS) by quantitation of F-actin, clustering of tumor antigen, polarization of lytic granules (LGs), and distribution of key signaling molecules within the IS. Long-term killing capability, but not secretion of conventional cytokines or standard 4-h cytotoxicity, correlates positively with the quality of the IS in two different CAR T-cells that share identical antigen specificity. Xenograft model data confirm that the quality of the IS *in vitro* correlates positively with performance of CAR-modified immune cells *in vivo*. Therefore, they propose that the quality of the IS predicts the effectiveness of CAR-modified immune cells, which provides a novel strategy to guide CAR therapy. Yingmei Luo et al. (Luo et al., 2022) develop IL-12 nanostimulant-engineered CAR T-cell (INS-CAR T) biohybrids for boosting antitumor immunity of CAR T cells *via* immunofeedback. IL-12 is responsively released from INS-CAR T biohybrids in presence of the increased thiol groups on cell-surface triggered by tumor antigens. In return, released IL-12 obviously promotes the secretion of CCL5, CCL2, and CXCL10, which further selectively recruits and expands CD8^+^ CAR T-cells in tumors. Ultimately, the immune-enhancing effects of IL-12 nanochaperone significantly boost CAR T-cell antitumor capabilities, dramatically eliminated solid tumor and minimized unwanted side effects.

#### 5.2.3 Nanomedicines reduce immunosuppression in the tumor microenvironment

The efficacy of Adoptive Cell Therapy (ACT) for solid tumor is still mediocre. This is mainly because tumor cells can hijack ACT T-cells’ immune checkpoint pathways to exert immunosuppression in the tumor microenvironment. Immune Checkpoint Inhibitors such as anti-PD-1 (aPD1) can counter the immunosuppression, but the synergizing effects of aPD1 to ACT was still not satisfactory. (Chen et al., 2022) demonstrate an approach to safely anchor aPD1-formed nanogels onto T-cell surface *via* bio-orthogonal click chemistry before adoptive transfer. The spatial-temporal co-existence of aPD1 with ACT T-cells and the responsive drug release significantly improved the treatment outcome of ACT in murine solid tumor model. The average tumor weight of the group treated by cell-surface anchored aPD1 was only 18% of the group treated by equivalent dose of free aPD1 and T-cells. The result of this research indicates that technology can be broadly applicable in ACTs employing natural or Chimeric Antigen Receptor (CAR) T-cells. In addition, the exploitation of embedded protein-targeted nanoliposomes (Xie et al., 2021) and γδ-T cell therapies (Man et al., 2019) would be beneficial to reduce immunosuppression of the tumor microenvironment, non-invasive cell tracking approaches and increased targeting to tumor sites.

#### 5.2.4 Nanomedicines improve T-cell transport

Laura Wayteck et al. (Wayteck et al., 2016) conclude that many barriers need to be overcome in route to the tumor by developing a nanoparticle that can couple to the surface of CD8 (+) T-cells. Verified that liposomes reversibly attach to the surface of cytotoxic T lymphocytes *via* reduction-sensitive coupling. The activation state of the T-cell and liposome composition was shown to strongly influence the up-sampling efficiency. Loading cells with liposomes did not impair the activation status of the T-cells and the liposome composition are shown to strongly influence the loading efficiency. Loading the cells with liposomes does not compromise T-cell functionalities like proliferation and cytolytic function.

#### 5.2.5 Stimulation of intrinsic T-cell activity

Karlo Perica’s team (Perica et al., 2014) manufactured Artificial antigen presenting cells (aAPC) based on two types of nanoscale particle platforms: biocompatible iron-dextran paramagnetic particles (50–100 nm in diameter) and avidin-coated quantum dot nanocrystals (∼30 nm). Nanoscale aAPC induced antigen-specific T-cell proliferation from mouse splenocytes and human peripheral blood T-cells. When injected *in vivo*, both iron-dextran particles and quantum dot nanocrystals enhanced tumor rejection in a subcutaneous mouse melanoma model. This is the first description of nanoscale aAPC that induce antigen-specific T-cell proliferation *in vitro* and lead to effective T-cell stimulation and inhibition of tumor growth *in vivo*. This team of investigators have manufactured two types of nanoscale particle platform-based aAPCs and demonstrates that both iron-dextran particles and quantum dot nanocrystals enhance tumor rejection in a melanoma model, providing the first description of nanoscale aAPCs that lead to effective T-cell stimulation and inhibition of tumor growth.

#### 5.2.6 Ways to monitor the therapeutic effectiveness of CAR-T cell therapy

Adoptive cell transfer of targeted chimeric antigen receptor (CAR) T-cells has emerged as a highly promising cancer therapy. The pharmacodynamic action or CAR T-cells is closely related to their pharmacokinetic profile; because of this as well as the risk of non-specific action, it is important to monitor their biodistribution and fate following infusion. Stefan Harmsen (Harmsen et al., 2021) developed a dual-modal PET/near infrared fluorescent (NIRF) nanoparticle-based imaging agent for non-genomic labeling of human CAR T cells. Since the PET/NIRF nanoparticles did not affect cell viability or cytotoxic functionality and enabled long-term whole-body CAR T-cell tracking using PET and NIRF in an ovarian peritoneal carcinomatosis model, this platform is a viable imaging technology to be applied in other cancer models.

Combination with nanotechnology, CAR-T cells can construct a lentiviral expression vector and complete the culture of a complete CAR-T cell expansion system. Clinical studies (Hockemeyer et al., 2011) have shown that combining nanotechnology with CAR-T can provide better remission and treatment for highly recurrent pediatric acute lymphoblastic leukemia. Meanwhile, patients with poor prognosis B-cell malignancies can be effectively treated by CAR-T cell redirected immunization (Ren et al., 2017). Due to the frequency of chemotherapy resistance and relapse, Leukemia can lead to certain adverse effects, such as severe acute tissue necrosis and other symptoms (Eyquem et al., 2017). Combining with nanotechnology and CAR-T can precisely screen the best target antigens, significantly reduce CAR- T-cell resistance, and significantly improve the remission rate of leukemia patients. For treating acute lymphoblastic leukemia, abandoning traditional chemotherapy and opting for nanotechnology and CAR-T can fundamentally address the problem of overload tumor in drug-resistant patients. Therefore, nanotechnology combining with CAR-T has a broad application prospect in the treatment of malignant tumors.

## 6 Conclusion and outlook

In recent years, the rapid development of nanomedicine provides new insights to cancer immunotherapy. The most obvious advantage of nanoparticles is tunability. They can be designed in various sizes and shapes. Being modified with targeting molecules or loading various drugs shows the ability of targeted delivery and therapeutic delivery simultaneously. Nanomedicines can also be targeted for immunotherapy applications based on different needs because of different materials properties, such as high targeting requirements, transporting small polarities, poor solubility, not easily encapsulated, and unable to overcome biological barrier situations. Finally, compared to low molecular weight immunomodulators, nanoscale immunomodulators control pharmacokinetic behavior and have the potential to enhance immune activation through synergistic effects due to their unique size effect and co-loading capacity. Indeed, the presence of multiple functional domainsovercome barriers to effective immunotherapy in solid tumors. This paper introduces the characteristics and advantages of immunotherapy, nanomedicines and their current research status in immunotherapeutic approaches, focusing on reviewing the application and prospects of various types of nanoparticles in CAR-T therapy for tumor treatment, learning about the advantages of nanomedicines in immunotherapy and preparing for the development of more nanomedicines in CAR-T therapies for oncology.

Currently, CAR-T therapies mainly work in hematologic tumors and lymphomas. It has much less effect in solid tumors with toxicity. This variation is caused by various factors, For example, multiple cells and molecules with different immunosuppressive effects are appeared in solid tumors; the level of antigen expression in solid tumor cells varies between tumor types and different stages; and antigens in solid tumors are generally expressed in small amounts in other sites, such as heart, lung, and liver, accompanying by off-target effects. Therefore, nanoparticle immunotherapy can be used in combination therapies to address the limitations solid tumors treatments, including chemotherapy, radiotherapy, and photodynamic therapy. For example, compared to immunotherapy, radiotherapy combing with immunotherapy significantly improves the efficiency of patient treatment in clinical trials, which overturning the traditional view that radiotherapy kills immune cells and has an immunosuppressive effect. This suggests a synergistic effect of radiotherapy and immunotherapy (Ai et al., 2020).

Partial radiotherapy activates the immune system, inducing immune cells to attack tumor cells outside the radiotherapy area. It is rare for clinical radiotherapy but immunotherapy can enhance the immune-inducing effects of radiotherapy and increase the incidence of distant effects (Wang 2020). Although the synergistic effects of combination regimens have been confirmed through various phase III clinical studies, clinicians still have many questions about their application, especially some controversial issues needing more valid information. With a better understanding of cancer immunology and nanomedicine, efforts are still needed to optimize the properties of nanoparticles to assess their potential risks before translating them into clinical practice. More effective nano-types and nanoparticles will transform cancer immunotherapy in the near future.
